# Non-Specific Lipid Transfer Protein Amb a 6 Is a Source-Specific Important Allergenic Molecule in Ragweed Pollen

**DOI:** 10.3390/ijms25126513

**Published:** 2024-06-13

**Authors:** Manuela Grijincu, Gabriela Tănasie, Lauriana-Eunice Zbîrcea, Maria-Roxana Buzan, Tudor-Paul Tamaș, Monica-Daniela Cotarcă, Ioan Huțu, Elijahu Babaev, Frank Stolz, Yulia Dorofeeva, Rudolf Valenta, Virgil Păunescu, Carmen Panaitescu, Kuan-Wei Chen

**Affiliations:** 1 Center of Immuno-Physiology and Biotechnologies, Department of Functional Sciences, Victor Babeș University of Medicine and Pharmacy, 300041 Timișoara, Romania; 2 OncoGen Center, Pius Brînzeu County Clinical Emergency Hospital, 300723 Timișoara, Romania; 3 Horia Cernescu Research Unit, Faculty of Veterinary Medicine, University of Life Sciences “King Michael I of Romania”, 300645 Timișoara, Romania; 4 Biomay AG, Vienna Competence Center, 1220 Vienna, Austria; 5 Department of Pathophysiology and Allergy Research, Division of Immunopathology, Center of Pathophysiology, Infectiology and Immunology, Medical University of Vienna, 1090 Vienna, Austria; 6 NRC Institute of Immunology FMBA of Russia, 115478 Moscow, Russia; 7 Department of Clinical Immunology and Allergy, Sechenov First State Medical University, 119991 Moscow, Russia; 8 Karl Landsteiner University of Health Sciences, 3500 Krems, Austria

**Keywords:** allergy, common ragweed, nsLTP, Amb a 6, recombinant allergen, ImmunoCAP, basophil activation test

## Abstract

Pollen from common ragweed is an important allergen source worldwide and especially in western and southern Romania. More than 100 million patients suffer from symptoms of respiratory allergy (e.g., rhinitis, asthma) to ragweed pollen. Among the eleven characterized allergens, Amb a 6 is a non-specific lipid transfer protein (nsLTP). nsLTPs are structurally stable proteins in pollen and food from different unrelated plants capable of inducing severe reactions. The goal of this study was to produce Amb a 6 as a recombinant and structurally folded protein (rAmb a 6) and to characterize its physicochemical and immunological features. rAmb a 6 was expressed in *Spodoptera frugiperda Sf9* cells as a secreted protein and characterized by mass spectrometry and circular dichroism (CD) spectroscopy regarding molecular mass and fold, respectively. The IgE-binding frequency towards the purified protein was evaluated using sera from 150 clinically well-characterized ragweed-allergic patients. The allergenic activities of rAmb a 6 and the nsLTP from the weed *Parietaria judaica* (Par j 2) were evaluated in basophil activation assays. rAmb a 6-specific IgE reactivity was associated with clinical features. Pure rAmb a 6 was obtained by insect cell expression. Its deduced molecular weight corresponded to that determined by mass spectrometry (i.e., 10,963 Da). rAmb a 6 formed oligomers as determined by SDS-PAGE under non-reducing conditions. According to multiple sequence comparisons, Amb a 6 was a distinct nsLTP with less than 40% sequence identity to currently known plant nsLTP allergens, except for nsLTP from *Helianthus* (i.e., 52%). rAmb a 6 is an important ragweed allergen recognized by 30% of ragweed pollen allergic patients. For certain patients, rAmb a 6-specific IgE levels were higher than those specific for the major ragweed allergen Amb a 1 and analysis also showed a higher allergenic activity in the basophil activation test. rAmb a 6-positive patients suffered mainly from respiratory symptoms. The assumption that Amb a 6 is a source-specific ragweed allergen is supported by the finding that none of the patients showing rAmb a 6-induced basophil activation reacted with Par j 2 and only one rAmb a 6-sensitized patient had a history of plant food allergy. Immunization of rabbits with rAmb a 6 induced IgG antibodies which strongly inhibited IgE binding to rAmb a 6. Our results demonstrate that Amb a 6 is an important source-specific ragweed pollen allergen that should be considered for diagnosis and allergen-specific immunotherapy of ragweed pollen allergy.

## 1. Introduction

Common ragweed (*Ambrosia artemisiifolia*) pollen has been identified as an important source of allergens in the whole world causing allergy in more than 100 million patients [1,2,3]. Ragweed occurs as an endemic weed in certain regions of the world, for example, in the United States, but it has also been introduced to certain parts of Europe (e.g., Lyon, France; Hungary; northern Italy; western and southern Romania; Ukraine; Russia; and Turkey) [4,5,6,7,8,9,10,11]. Furthermore, ragweed plants have spreadto other continents, including Asia, Australia, Africa, and South America [12]. Ragweed-allergic patients experience allergic rhinitis and asthma during the pollen season in August–September, which worsen under certain conditions by increasing allergen or pollen content in the atmosphere, i.e., storms, urbanization, and pollution [2,13,14,15]. Currently, eleven ragweed pollen allergens have been described, among which Amb a 1 and Amb a 11 are classified as major allergens according to the frequency of IgE recognition [16,17]. Four allergens belong to known allergen families, profilin Amb a 8, polcalcins Amb a 9 and 10, and non-specific lipid transfer protein (nsLTP) Amb a 6, which may show varying degrees of IgE cross-reactivity [16]. Patients sensitized to ragweed pollen were found to have complex IgE sensitization patterns [18]. Patients tended to report more symptoms when sensitized to pan-allergens in addition to Amb a 1 [18,19]. Importantly, the allergenic activity of the individual ragweed allergens needs to be evaluated in more detail because the frequency of IgE recognition does not take into consideration this important parameter for the clinical relevance of a given allergen [20,21,22].

Among the ragweed allergens, nsLTPs are pathogenesis-related proteins belonging to the prolamin superfamily [23]. The nsLTPs are stabilized by eight cysteine residues forming a hydrophobic cavity able to bind fatty acids and confer stability to both heat and proteolytic digestion [23,24]. The structural stability is an important contributor to nsLTP allergenicity, especially in food allergy, by exposing large allergen fragments to the immune system, thereby facilitating IgE sensitization via the gastrointestinal tract [25,26].

nsLTPs have been studied initially as major allergens in peach (Pru p 3) and wall pellitory (*Parietaria judaica*) (Par j 1 and Par j 2) [27,28]. As major allergens, nsLTPs were found to trigger a wide range of symptoms, including asthma and anaphylaxis, sometimes requiring the presence of certain cofactors [27,28,29]. The mugwort nsLTP Art v 3 has been extensively studied regarding the role in pollen–food allergy syndrome, due to possible cross-reactivity with peach Pru p 3 [30], but more recent analyses indicate that there is not much cross-reactivity between pollen and plant food nsLTPs [31]. No symptoms of food allergy have been mentioned for patients sensitized to mugwort pollen and Art v 3 [32]. The high ragweed pollen load in Romania and the ability of nsLTPs to trigger severe symptoms underline the importance of investing the role of Amb a 6 in ragweed pollen allergy. Two different Amb a 6 isoforms, named Ra6A and Ra6B, were first identified and isolated from ragweed pollen in 1983 [33]. The two isoforms were immunologically indistinguishable, had an IgE frequency of 21%, and sensitization was associated with HLA-DR5 among ragweed pollen-allergic patients [33,34]. The potential cross-reactivity, allergenicity, and association with clinical features have not been extensively investigated. Allergen characterization requires the availability of pure, measurable amounts of protein, facilitated by recombinant protein production [35]. Since one study reported that Amb a 6 was successfully produced in an eukaryotic expression system, *Spodoptera frugiperda Sf9* cells were chosen for Amb a 6 expression [36].

The physicochemical features of the purified protein were evaluated and the protein was then used to test IgE recognition frequency and allergenic activity among clinically well-characterized ragweed pollen-allergic patients. Thus IgE recognition of Amb a 6 could be investigated in association with clinical phenotypes of allergy.

## 2. Results

### 2.1. Recombinant Expression, Purification, and Biochemical Characterization of Amb a 6

**Figure 1:**
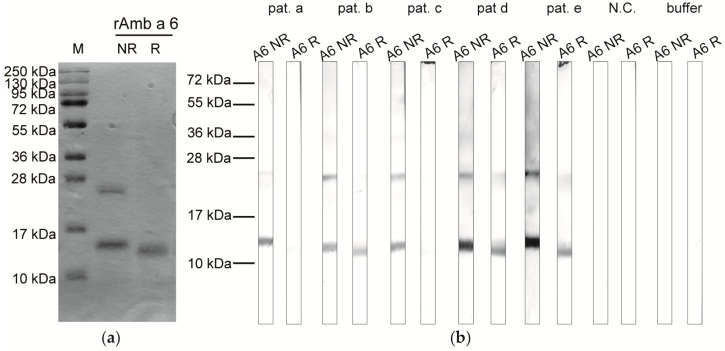
Overview of recombinant allergen production: (**a**) SDS-PAGE with the purified rAmb a 6 separating under non-reducing (NR) and reducing (R) conditions; (**b**) IgE binding towards rAmb a 6 (A6) separated under NR and R conditions using serum from five ragweed-allergic patients, one non-allergic subject (N.C.), and buffer used as control. A molecular weight marker is shown on the left for both SDS-PAGE and immunoblot.

rAmb a 6 was obtained as a pure protein with a yield of 1 mg of protein from 1 L of cell culture (2 × 10^6^ cells/mL). When separated by SDS-PAGE under non-reducing (NR) conditions, rAmb a 6 formed two bands at approximately 13–14 kDa and 27 kDa, respectively. The bands seem to represent a monomer and dimer, which migrated at a higher molecular weight than calculated according to the sequence, most likely due to the alkaline isoelectric point (pI) of rAmb a 6 causing retention in the gel [37,38] (Figure 1a). Under reducing (R) conditions, the low molecular weight band migrated a bit lower than under NR conditions (Figure 1a). Most patients’ sera showed weaker or no IgE binding towards the allergen separated under R conditions, whereas IgE binding towards both bands was observed after the allergen had been separated under NR conditions (Figure 1b). The fact that the band corresponding to a possible dimer disappeared or was weaker after the allergen had been separated under R conditions indicates that the dimer formation was likely due to intermolecular disulfide bonds.

**Figure 2:**
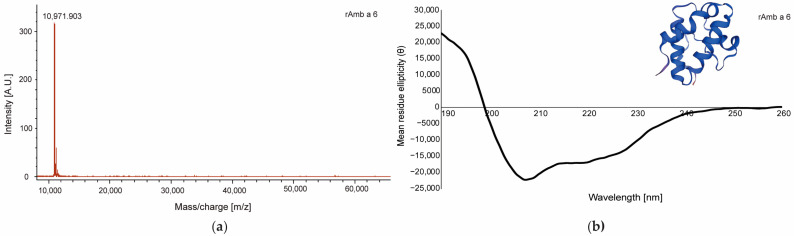
Physicochemical characterization of the recombinant allergen Amb a 6 (rAmb a 6): (**a**) MALDI-ToF mass spectrum for rAmb a 6. The mass/charge ratio is shown on the *x*-axis; (**b**) Far UV spectrum of rAmb a 6. The mean residue ellipticity θ (deg × cm^2^/dmol) is shown on the *y*-axis and the wavelength (nm) is shown on the *x*-axis; the 3D model of Amb a 6, excluding the signal peptide is also shown [40].

In MALDI-ToF, the mass of rAmb a 6 was 10,971.9 Da, which corresponds to the calculated molecular weight based on the amino acid sequence determined in the Protparam Expasy tool as 10,963.61 Da [39] (Figure 2a). The far UV circular dichroism spectrum of rAmb a 6 revealed a predominantly α-helical structure, indicated by the minimum between 200 and 210 nm (Figure 2b). The proportion of secondary structures calculated in DiChroWeb showed that the protein consisted of 54% α-helix structures, 8% β-sheets, 13% turns, and 24% unordered structures. The predominantly α-helical structure appeared also in the 3D model generated in Expasy, in which Amb a 6 consisted of four α-helix chains (Figure 2b) [40]. The protein sequence also indicated the existence of an N-glycosylation site in position 38–41 according to the Prosite scan, although the mass spectrometry result did not show any relevant glycosylation [41].

**Figure 3:**
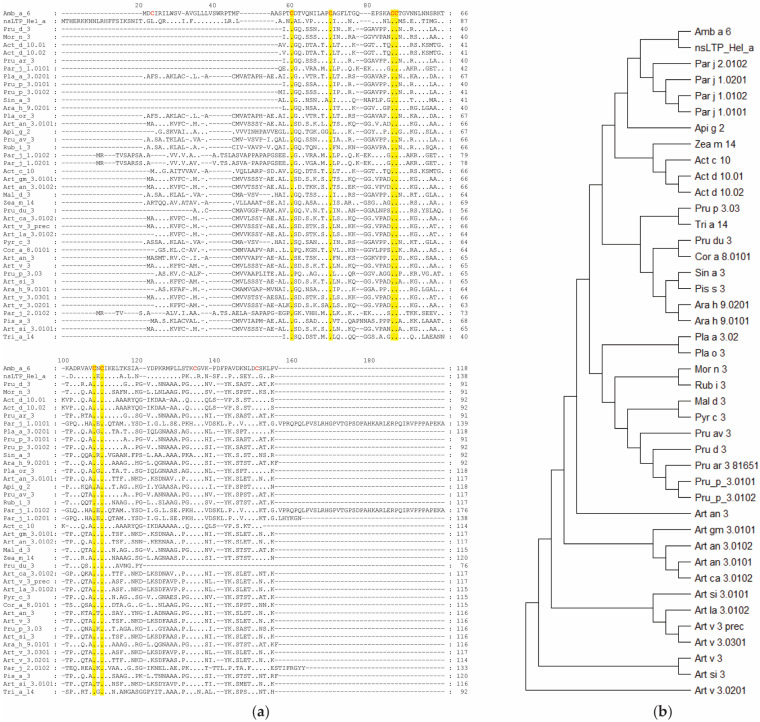
Homology of Amb a 6 with other known nsLTP allergens: (**a**) Sequence alignment of Amb a 6 with 41 other similar allergenic nsLTPs, with amino acids conserved among all nsLTPs highlighted in yellow and cysteine residues of Amb a 6 evidenced in bold red text; (**b**) Maximum parsimony phylogenetic tree of the allergenic proteins detected by NCBI Blast.

The sequence alignment of Amb a 6 with other allergenic nsLTPs (Table S1) showed that the only conserved amino acids were six cysteine residues (Figure 3a). The phylogenetic tree of the sequences generated by maximum parsimony showed that Amb a 6, the nsLTP from sunflower (Hel a), and *Parietaria* nsLTPs formed a cluster although the sequence identities of the proteins were very low. Another cluster was formed by the different isoforms of nsLTPs from different mugwort species. Similarly, the allergenic nsLTPs from different *Rosacea* species clustered, and peanut, pea, and mustard nsLTPs formed another separate cluster (Figure 3b). Amb a 6 had the highest sequence identity with the nsLTP from sunflower (51%), while sequence identities with the other allergenic nsLTPs were very low (i.e., below 40%). The lowest sequence identity with Amb a 6 and nsLTPs was recorded for Tri a 14 from wheat (i.e., 27%) (Table S2).

### 2.2. IgE Reactivity towards rAmb a 6

**Figure 4:**
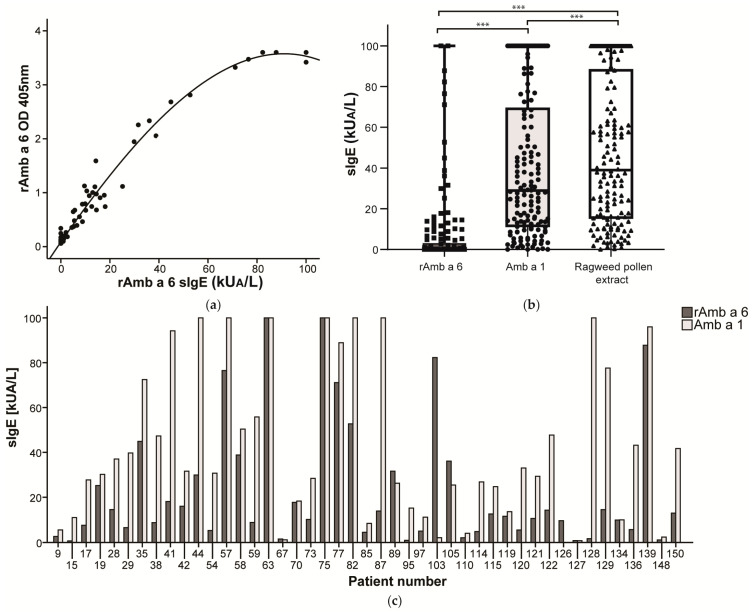
IgE recognition of rAmb a 6: (**a**) Correlation between the optical densities (OD) obtained for rAmb a 6 in IgE ELISA and the specific IgE levels determined by ImmunoCAP for 150 ragweed pollen-allergic patients; (**b**) Specific IgE reactivity towards rAmb a 6, Amb a 1, and ragweed pollen extract for 150 ragweed-allergic patients. In the boxplot, the line shows the median, the box represents the 25th and 75th percentile, and the whiskers show the minimum and maximum values. Statistically significant differences are marked as *** *p* < 0.001; (**c**) Specific IgE levels towards rAmb a 6 and Amb a 1 for 45 patients reactive towards rAmb a 6 in ImmunoCAP.

The frequency of IgE binding of rAmb a 6 was tested both by IgE ELISA and by quantitative ImmunoCAP measurements. Out of 150 patients allergic to ragweed pollen, 47 patients (31.3%) were positive in ELISA for IgE against rAmb a 6, while in ImmunoCAP measurements 45 patients (30%) had sIgE values above 0.35 kU_A_/L. Out of the 150 patients, 42 were positive in both methods, while 5 were positive only in ELISA and 3 patients were over the diagnostic threshold in ImmunoCAP. The values for optical density (OD) in ELISA and specific IgE (sIgE) in ImmunoCAP were significantly correlated (ρ = 0.689, *p* < 0.001, *n* = 150) (Figure 4a). The quantitative ImmunoCAP sIgE levels were used for further comparisons with ragweed pollen and Amb a 1 sIgE in the Wilcoxon test. The sIgE levels towards rAmb a 6 were significantly lower than those towards Amb a 1 (*p* < 0.001) and towards ragweed pollen (*p* < 0.001) (Figure 4b). This difference remained when the sIgE levels were compared among rAmb a 6-positive patients (comparison of rAmb a 6 with Amb a 1, *p* < 0.001; with ragweed pollen *p* < 0.001, *n* = 45). However, six patients had higher sIgE levels towards rAmb a 6 than towards the major allergen Amb a 1 (Pat. 67, 89, 103, 105, 126, 127) and two patients had similar sIgE levels towards both allergens (Pat. 63, 75) (Figure 4c, Table S3).

### 2.3. Rabbit Antibodies Obtained by Immunization with rAmb a 6 Inhibit Patients’ IgE Binding to rAmb a 6

The rabbit serum showing the highest IgG reactivity towards rAmb a 6 was tested regarding its ability to inhibit allergic patients’ IgE binding towards rAmb a 6. The inhibition achieved with rabbit rAmb a 6-specific serum ranged between 37% and 95% with an average of 74% (Table 1). There was almost no inhibition with rabbit rAmb a 4-specific control serum, with the inhibition percentages ranging between 0 and 5% and an average of 1.9% IgE binding inhibition towards rAmb a 6 (Table 1).

### 2.4. Allergenic Activity of rAmb a 6 and the nsLTP from Parietaria judaica Pollen, Par j 2, and Association of Amb a 6 Sensitization with Clinical Features

**Figure 5:**
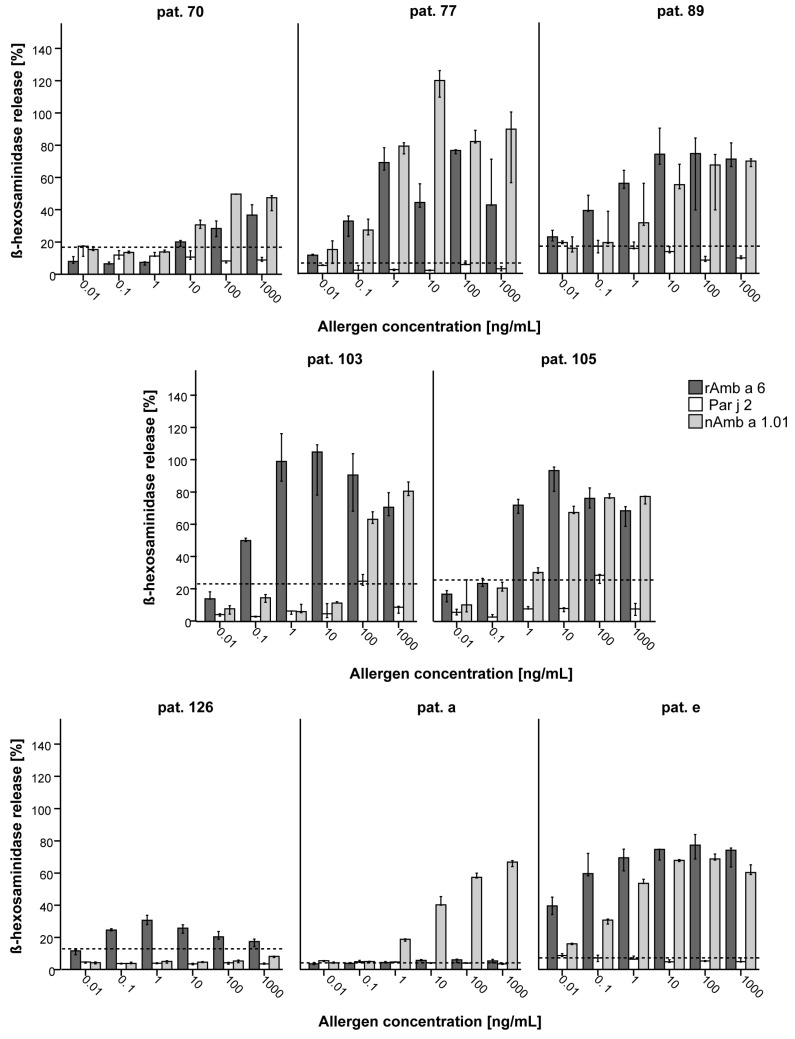
Mediator release assay towards rAmb a 6, nAmb a 1.01, and Par j 2 using serum from ragweed-allergic patients. The results are shown as the percentage of total release, bar charts represent the median release, and the error bars show the 95% confidence interval. The dashed line indicates the degranulation obtained from cells with serum without allergen (cut-off). Allergen concentrations (ng/mL) are displayed on the *x*-axes, with the *y*-axes showing the percentages of total release (%).

A considerable proportion of rAmb a 6-sensitized patients showed equal to higher IgE levels specific for rAmb a 6 than for the major ragweed allergen Amb a 1. Therefore, the allergenic activity of rAmb a 6 was evaluated in basophil activation tests (Figure 5). A mediator release assay with serum from rAmb a 6-positive patients was performed, showing lower or comparable sIgE values for rAmb a 6 and Amb a 1 (Table S3). rAmb a 6 induced up to 100% of β-hexosaminidase release from IgE-loaded cells from one patient at 100 ng/mL and 10 ng/mL, reaching up to 50% mediator release even at the second lowest dilution (Pat. 103). The overall Amb a 6 reactivity was comparable with the reactivity towards nAmb a 1.01 or slightly higher for certain patients, which also showed higher levels of sIgE in ImmunoCAP (Pat. 89, 103, 105). For one patient, which was positive for ragweed pollen in ImmunoCAP, but negative for nAmb a 1.01, rAmb a 6 induced mediator release of around 30% at 1 ng/mL (Pat. 126). The nsLTP from *Parietaria judaica* pollen, Par j 2, did not induce mediator release for any of the rAmb a 6-sensitized patients’ sera tested, demonstrating that rAmb a 6 and Par j 2 do not share IgE epitopes (Figure 5).

**Figure 6:**
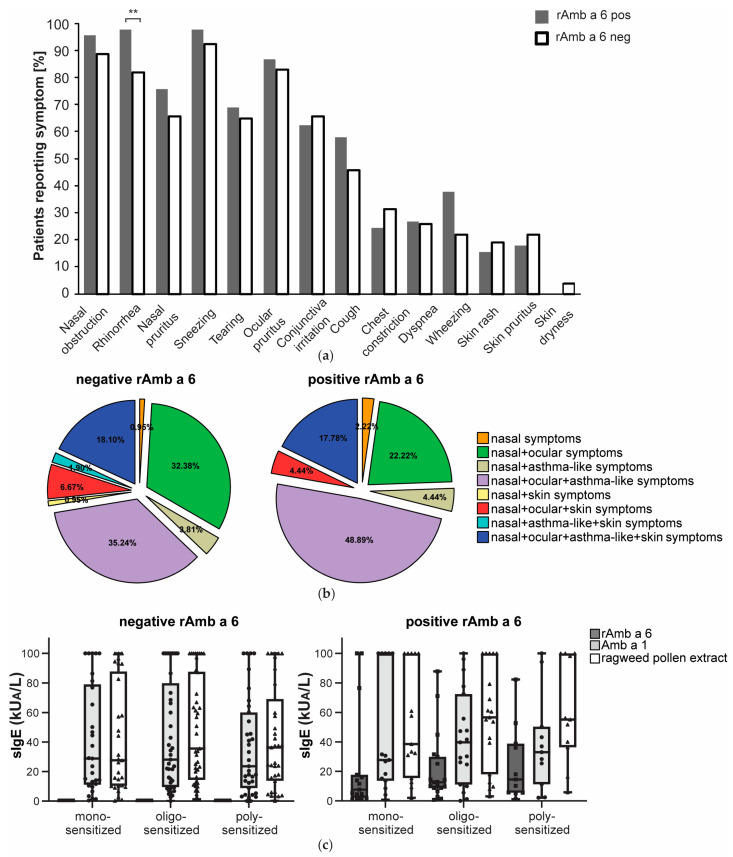
Lack of clear association between reactivity to rAmb a 6 and the clinical features of ragweed-allergic patients: (**a**) Allergy-related symptoms reported by patients positive towards rAmb a 6 in ImmunoCAP compared to those which were negative in ImmunoCAP. Statistically significant results are marked as ** *p* < 0.01; (**b**) Symptom types reported by patients positive and negative towards rAmb a 6 in ImmunoCAP; (**c**) Boxplots showing sIgE levels towards rAmb a 6 in monosensitized, oligo-, and polysensitized ragweed-allergic patients according to SPT among rAmb a 6-positive and -negative patients. In the boxplots, the horizontal lines show the median, the box represents the 25th and 75th percentile, and the whiskers show the minimum and maximum values.

Since the cohort of patients included in the study was meticulously characterized regarding clinical parameters, the IgE reactivity profiles could be associated with clinical phenotypes. Table S3 contains the results of IgE antibody measurements specific for rAmb a 6, Amb a 1, and ragweed pollen extract. Information regarding sensitization to allergen sources other than ragweed pollen and allergic symptoms to other allergen sources is also included in Table S3. The presence/absence of certain symptoms and reactivity towards rAmb a 6 were tested in a Fisher exact test. Overall, rhinorrhea was more frequently reported amongst rAmb a 6-positive patients than amongst those who were negative (44/45 (97.7%) vs. 86/105 (81.9%), *p* = 0.008). Wheezing was also reported relatively more often amongst patients positive towards rAmb a 6 (17/45 (37.7%)) than amongst those negative towards rAmb a 6 (23/105 (21.9%)). However, the difference did not reach statistical significance (*p* = 0.06) (Figure 6a). When clustering the symptoms into symptom types, asthma-like symptoms in addition to nasal and ocular symptoms were reported more frequently among patients sensitized to rAmb a 6 (48.9%) than among those negative towards rAmb a 6 (35.2%) (*p* = 0.117). A higher proportion of rAmb a 6-negative patients reported only nasal and ocular symptoms (32.4%) than rAmb a 6-positive patients (22.2%) (*p* = 0.210) (Figure 6b). A slightly higher proportion of rAmb a 6-reactive patients reported more than two symptom types (32/45 (71%)) than among rAmb a 6-negative patients (65/105 (62%)) (*p* > 0.05) (Figure 6b). The sIgE levels towards rAmb a 6, Amb a 1, and ragweed pollen extract were highly correlated (*p* < 0.01) but did not correlate with the duration since allergy onset. The overall sIgE levels were the highest towards ragweed pollen extract, followed by Amb a 1, and then rAmb a 6 (*p* < 0.01). In ragweed pollen-monosensitized patients, Amb a 1 sIgE levels did not differ significantly from ragweed pollen extract sIgE levels (*p* = 0.071), whereas among rAmb a 6-reactive polysensitized patients, rAmb a 6 sIgE did not differ significantly from Amb a 1 sIgE levels (*p* = 0.091) (Figure 6c).

Patients were asked whether they experienced allergy symptoms to plant foods and the reactivity towards foods was evaluated by skin prick testing or ImmunoCAP measurements. Therefore, possible associations between IgE sensitization to rAmb 6 and plant–food allergy potentially caused by cross-reactive nsLTPs could be investigated (Table S3). Only 1 out of the 50 Amb a 6-sensitized patients (i.e., Patient 128) (ELISA and/or ImmunoCAP) suffered from allergy to hazelnuts, whereas the other 3 patients with symptoms of plant food allergy (i.e., Patients 39, 46, 78) were negative for rAmb a 6. Thus, it was unlikely that IgE sensitization to Amb a 6 triggered IgE-mediated allergy to nsLTP-containing plant foods in the tested patients.

## 3. Discussion

The ragweed nsLTP Amb a 6 was discovered as an allergen 40 years ago [33]. A detailed characterization of an allergen for component-resolved diagnosis and personalized therapeutic approaches requires the availability of a homogenous, pure, and defined allergen preparation [35]. The goal of our study was to perform a detailed characterization of the Amb a 6 allergen regarding its IgE binding frequency and allergenic activity and the possible association of its immunological characteristics with clinical data obtained in a representative population of ragweed allergic patients.

For this purpose, recombinant Amb a 6 was produced in a eukaryotic expression system, i.e., in insect cells. Eukaryotic expression in insect cells was chosen because previous studies have found for the nsLTP from *Parietaria judaica* pollen, i.e., Par j 2, that the insect cell-expressed allergen exhibited a higher IgE reactivity and allergenic activity [42]. Highly pure and folded rAmb a 6 was obtained exhibiting the expected molecular mass according to its amino acid sequence. The protein contained nine cysteine residues of which eight seemed to have assembled properly to stabilize the protein which consisted mainly of α-helical secondary structure as determined by far UV CD analysis. This mostly α-helical structure with an unstructured C-terminus was described as characteristic of nsLTPs, confirming the nsLTP structure of the produced protein [10]. It is a limitation of this study that it did not include the near UV CD analysis of rAmb a 6, since it was difficult to obtain highly concentrated folded protein and rAmb a 6 contained a hexa-histidine tag which could influence the near UV CD results. SDS-PAGE demonstrated that rAmb a 6 consisted of a monomeric form and a dimer. The abundant dimer observed under non-reducing SDS-PAGE disappeared under reducing conditions and therefore seemed to be due to the formation of intermolecular disulfide bonds. The separation of rAmb a 6 by native PAGE was not possible, probably due to the alkaline isoelectric point. A weaker IgE recognition was found for the dimer in immunoblot. This may be due to the fact that the dimer band was much weaker on SDS-PAGE, indicating that the dimer occurs in smaller amounts in the preparation. However, it is also possible that IgE binding sites were blocked by the dimer formation. Similar findings have been reported for the hazelnut (Cor a 8) and mustard (Sin a 3) nsLTPs; in both cases, the dimer showed less or no IgE recognition [43,44]. The IgE recognition frequency of rAmb a 6 among 150 ragweed-allergic patients was 30% which was in good agreement with IgE reactivity observed for the natural allergen indicating that the recombinant protein was obtained in an authentic IgE-reactive form [33]. Based on this IgE recognition frequency, rAmb a 6 cannot be classified as a major allergen. However, our data indicate that it is an important allergen since Amb a 6-specific IgE levels and the allergenic activity in basophil activation experiments were high in a considerable number of the IgE-positive patients. The fact that Amb a 6 induced strong IgE-dependent basophil activation provides additional support for the allergenic activity of the recombinant allergen. Accordingly, Amb a 6 can be considered an important component for the molecular diagnosis of ragweed pollen allergy and for the development of molecular allergen-specific immunotherapy (AIT) formulations for ragweed pollen allergy. Regarding respiratory allergy, the detailed analysis of the symptoms among rAmb a 6-positive and -negative patients has not shown a difference regarding association with a certain symptom, except rhinorrhea. However, a higher proportion of rAmb 6-positive patients tended to report asthma-like symptoms (71% vs. 59%), especially wheezing which was more frequently reported. This finding hints towards the clinical relevance of Amb a 6, highlighting its role in ragweed pollen allergy. Additionally, patients sensitized to nsLTP more frequently reported more than two symptom types, suggesting that Amb a 6 in addition to Amb a 1 is important for patients reporting asthma with rhino-conjunctivitis [18]. The levels of sIgE towards Amb a 6 did not differ from Amb a 1 sIgE for polysensitized nsLTP-reactive patients. This finding is similar to that by Barber et al., claiming that minor allergens become important in areas with high exposure to certain allergen sources [45]. Thus, the present study underlines the importance of a personalized approach to nsLTP allergy diagnosis, considering the patients’ exposure and other sensitization in addition to serum IgE measurements.

nsLTPs from various plants have been described as allergens in pollen and somatic plant tissues, thus causing respiratory and/or plant food allergy [37,38]. The importance of IgE cross-reactivity between nsLTPs from pollen and plant food is a matter of discussion. Some studies suggest there may be relevant IgE cross-reactivity [30], whereas other studies and meta-analyses suggest that there is not much relevant IgE cross-reactivity between pollen and plant food nsLTPs or between different pollen nsLTPs [31]. Data from our study would rather indicate that Amb a 6 is a genuine and source-specific allergen in ragweed pollen without relevant IgE cross-reactivity to other nsLTP allergens. First of all, the multiple sequence alignment of Amb a 6 with other nsLTP allergens showed that Amb a 6 had only a very low sequence identity with other nsLTPs which is below a percentage that would be expected to yield strong IgE cross-reactivity [46]. Only the nsLTP from *Helianthus* had a sequence identity of 51%, whereas the sequence identities to other plant nsLTPs were all below 40%. Second, Par j 2, the major nsLTP allergen from *Parietaria judaica* pollen, did not induce basophil activation in any of the patients responding to rAmb a 6. Finally, the present study did not find any relevant evidence that the Amb a 6-sensitized patients suffered from plant food-induced allergy, indicating that Amb a 6 did not contribute to nsLTP-induced plant food allergy.

Therefore, Amb a 6 can be considered an important allergen molecule for the diagnosis of ragweed pollen allergy and as a marker allergen for genuine sensitization to ragweed which may help in the differential diagnosis of plant pollen and plant food polysensitization. Furthermore, Amb a 6 should be considered as an important molecule for ragweed pollen AIT. The latter assumption is supported by the fact that antibodies induced in rabbits by immunization with rAmb a 6 strongly inhibited allergic patients’ IgE binding to rAmb a 6. However, further studies will be necessary to evaluate the diagnostic usefulness of Amb a 6 in different populations of nsLTP-sensitized patients as has been studied for other nsLTPs [47]. In addition, it will be necessary to investigate and compare the IgE recognition frequency and allergenic activity of Amb a 6 with that of other ragweed pollen allergens to define the relevant molecules to be included in molecular AIT vaccines for the treatment of ragweed pollen allergy.

## 4. Materials and Methods

### 4.1. Serum from Ragweed-Allergic Patients

Sera were collected from 155 ragweed-allergic patients at an allergy clinical center in Timişoara, Romania, after informed consent was obtained. The diagnosis of ragweed pollen allergy was based on a positive skin prick test (SPT), the presence of specific IgE (sIgE) against ragweed pollen extract or Amb a 1, and an unambiguous recording of allergic symptoms during the ragweed pollen season. The serum samples were stored at −80 °C.

The patients were asked in detail about the symptoms experienced during the ragweed pollen season by questionnaire. These symptoms were classified based on the case history as follows: nasal symptoms (nasal obstruction, rhinorrhea, nasal pruritus, sneezing), ocular symptoms (tearing, ocular pruritus, conjunctiva irritation), asthma-like symptoms (cough, chest constriction, dyspnea, wheezing), skin symptoms (skin rash, skin pruritus, skin dryness). Sensitizations to additional allergen sources were evaluated by the recording of symptoms, SPT, and/or by measurement of serum sIgE. Patients were tested towards common inhalant allergen sources (ash, birch, hazel, timothy grass, wheat, rye, house dust mites, cat, dog, *Alternaria*, *Aspergillus*, *Candida*, *Cladosporium*, *Penicillium*) and food allergens (potato, apple, banana, lemon, orange, peanuts, hazelnuts, walnuts, wheat flour, barley flour, egg white, egg yolk, crustaceans, salmon, trout). Patients were considered monosensitized if the SPT was positive only towards ragweed pollen, oligosensitized if positive to at most two allergen sources other than ragweed, and polysensitized if positive to more than four allergen sources [48].

The usage of sera of ragweed-allergic patients in this project was approved by the Local Ethics Commission of Scientific Research of the Pius Brînzeu Emergency County Hospital, Timișoara (Ethical Statement No. 102, 10.01.2017). All experiments were performed following relevant guidelines and regulations.

### 4.2. Recombinant Allergen Production and IgE Immunoblotting

The sequence for Amb a 6 was retrieved from the Allergome database [49] (GenBank accession number AAB51146.1). A sequence blast was performed to identify similar allergens [50]. The identified sequences (Table S1) were then used to construct an identity matrix and a maximum parsimony phylogenetic tree in MEGA [51].

A nucleotide construct codon optimized for *Spodoptera frugiperda (Sf 9)* (Thermo Fisher Scientific, Waltham, MA, USA) insect cell expression without the signal peptide (from amino acids 1 to 25) and including a C-terminal hexa-histidine tag (103 AA, 10 kDa) was designed using the pTM1 vector via BamHI/SmaI sites (ATG Biosynthetics, Merzhausen, Germany).

The subcloning, baculovirus, and protein production were performed as described in [52]. The secreted protein was isolated and purified from the supernatant of *Sf 9* cells using a nickel (Ni^+^-NTA) agarose matrix (Qiagen, Hilden, Germany) according to the QIAexpressionist handbook [53]. Cells exposed to baculovirus for 96 h were centrifuged 4500× *g*, 5 min, 4 °C. The supernatant was dialyzed overnight against lysis buffer described in [53]. The matrix was equilibrated with the same buffer and then added to the supernatant and incubated for 2 h at 4 °C on a rocker at 20 rpm. The agarose bound supernatant was then transferred to a column which was rinsed with wash buffer as described in [53] to remove other proteins binding with lower affinity to the matrix. Finally, the protein was eluted using elution buffer containing 250 mM imidazole (Sigma Aldrich, St. Louis, MO, USA). Aliquots containing the highest allergen concentration according to SDS-PAGE were pooled and dialyzed to remove the NaCl and imidazole contained in the elution buffer. The purified protein was stored in 10 mM NaH_2_PO_4_, pH 6, final buffer at −20 °C.

Immunoblots of the protein running under reducing conditions (using sample buffer with ß-mercaptoethanol) and non-reducing conditions (sample buffer without ß-mercaptoethanol) were performed to verify the formation of oligomers and identification of IgE binding proteins. For blotting, 20 µg of rAmb a 6 (1:1.3 dilution with sample buffer) were separated on 14% SDS-PAGE and transferred onto a 0.2 µm nitrocellulose blotting membrane (ProtranTM, GE Healthcare Life Science, Chicago, IL, USA) at 150 mA for 1.5 h. The blots were cut into 0.5 cm strips containing rAmb a 6 and washed/blocked using buffer B (40 mM Na_2_HPO_4_, 0.6 mM NaH_2_PO_4_, pH 7.5, 0.5% Tween 20, 0.5% [*w*/*v*] Bovine serum albumin (BSA), 0.05% [*w*/*v*] NaN_3_). IgE binding proteins were detected by incubating the blotted strips with 1:10 diluted patient sera (labeled a–e) (Table S3) in buffer B. Bound IgE was detected with mouse anti-human IgE marked with alkaline phosphatase (AKP) (diluted 1:1000 in buffer B) (clone G7-26, BD Biosciences, Pharmingen, San Jose, CA, USA) and visualized after washing with AP buffer (100 mM Tris, 100 mM NaCl, 5 mM MgCl_2_) followed by addition of the detection buffer (10 mL AP buffer with 1.65 mg BCIP and 1.65 mg NBT) for 5 min.

### 4.3. Physicochemical Characterization

The determination of the mass of the purified proteins was performed by matrix-assisted laser desorption/ionization time-of-flight (MALDI-ToF) using a Microflex mass spectrometer (Bruker, Billerica, MA, USA) as previously described in [53].

Circular dichroism (CD) measurements were performed using a JASCO J-810 spectropolarimeter (Tokyo, Japan). The CD spectrum of purified rAmb a 6 was measured at room temperature at a concentration of 0.1 mg/mL using a rectangular quartz cuvette with 0.2 cm path length. Far ultraviolet (UV) spectra were recorded in the wavelength ranges between 190 and 260 nm with a resolution of 0.5 nm at a scan speed of 50 nm/min. Data from three measurements were averaged. The final spectra were baseline-corrected and the results were expressed as the mean residue ellipticity θ (deg × cm^2^/dmol) at a given wavelength. The secondary structure content of rAmb a 6 was calculated using the secondary structure estimation program DiChroWeb with the CDSSTR method [53,54].

### 4.4. IgE Reactivity towards rAmb a 6

Sera from 150 ragweed-allergic patients (labeled Pat. 1–150) were tested in ELISA against rAmb a 6. An amount of 0.5 µg of rAmb a 6 or rDer p 2 as a positive control for the calibration dissolved in PBS (136 mM NaCl, 2.6 mM KCl, 10 mM Na_2_HPO_4_, 1.7 mM KH_2_PO_4_, pH 7.4) was incubated overnight at 4 °C on 96-well MaxiSorp flat bottom plates (Thermo Fisher Scientific, Waltham, MA, USA). The plates were washed twice with PBS + 0.05% Tween (PBST) and then blocked with PBST + 3% BSA for 3 h at room temperature. One hundred microliters of 1:5 diluted patient serum in PBST + 0.5% BSA was added to the plates in duplicate and incubated overnight at 4 °C. After washing five times with PBST, horseradish peroxidase (HRP)-labeled polyclonal goat anti-human IgE (epsilon) antibody (SeraCare, Milford, MA, USA) diluted 1:2500 in PBST + 0.5% BSA was added at 100 µL/well and incubated for 45 min at 37 °C then 45 min at 4 °C. After five washes, the detection substrate was added containing 2,2′-Azino-bis(3-ethylbenzothiazoline-6-sulfonic acid) diammonium salt (ABTS) (Sigma Aldrich, St. Louis, MO, USA) in 60 mM citric acid, 77 mM Na_2_HPO_4_ × 2H_2_O, and 3 mM H_2_O_2_. The absorbance was measured at 405 nm with reference at 490 nm on a microplate reader (Tecan Infinite M200 Pro, Grödig, Austria) and readings at 25 min were used for further analysis. The cut-off was determined by using the mean optical density (OD) and the standard deviation of four ragweed non-allergic patients.

IgE binding in ImmunoCAP was performed by using commercially available Amb a 1 (w230) and ragweed pollen extract (w1) ImmunoCAPs, whereas rAmb a 6 was first bound to Streptavidin ImmunoCAP (o212, Thermo Fisher Scientific/Phadia, Uppsala, Sweden) as described in [19]. Specific IgE levels were measured on the Phadia 100 and Phadia 250 platform (Thermo Fisher Scientific). Specific IgE levels > 0.35 kU_A_/L were considered positive.

### 4.5. Induction of rAmb a 6-Specific Antibodies in Rabbits

rAmb a 6-specific polyclonal IgG antibodies were obtained by immunizing two White New Zealand (WNZ) rabbits with three doses of 200 µg of the recombinant allergen, using Freund’s complete adjuvant once and Freund’s incomplete adjuvant twice [55]. The titer of the IgG antibodies was determined in ELISA using eight serum dilutions (1:10^3^ to 1:10^10^) on 96-well MaxiSorp flat bottom plates (Thermo Fisher Scientific, Waltham, MA, USA) coated with 0.5 µg of rAmb a 6 and incubated overnight at 4 °C. The next day, the plates were washed with PBST and then blocked with PBST + 3% BSA for 2.5 h at room temperature. One hundred microliters of the eight serum dilutions (1:10^3^ to 1:10^10^) was then added to the plates and incubated overnight at 4 °C. Afterwards, the plates were washed five times with PBST, and HRP-labeled donkey anti-rabbit IgG (GE Healthcare, Chicago, IL, USA) diluted 1:2000 in PBST + 0.5% BSA was added and incubated first for 45 min at 37 °C and then for another 45 min at 4 °C. After another five washes with PBST, the substrate containing ABTS was added. The absorbance was measured as described in Section 4.4. The rabbit serum showing the highest Amb a 6-specific IgG reactivity (Figure S1) was used for further experiments.

### 4.6. Inhibition of Human IgE Binding to rAmb a 6 with Rabbit Amb a 6-Specific Serum and Detection of Potential Cross-Reactive Allergens

Competition ELISA to test the inhibition of human IgE binding to rAmb a 6 using rabbit rAmb a 6-specific serum was performed as described previously [53]. In short, plates were coated with 0.5 µg rAmb a 6 and incubated overnight. Plates were blocked the next day with PBST + 3% BSA and then incubated with 1:50 diluted rAmb a 6-specific rabbit serum (rabbit rAmb a 4-specific serum and serum before immunization were used as a negative controls) in PBST + 0.5% BSA and incubated overnight. The next day, 100 µL of 1:10 diluted patient serum in PBST + 0.5% BSA were added and incubated overnight. The detection procedure was identical to the one described in Section 4.4. Percent inhibition was calculated as follows:

inhibition [%] = (OD_pre_ − OD_imm_) × 100

where OD_pre_ is the optical density after pre-incubation with the rabbit pre-immune serum and OD_imm_ is the reactivity obtained after pre-incubation with rabbit immune serum.

### 4.7. Allergenic Activity of rAmb a 6

A mediator release assay was performed using humanized rat basophil leukemia cells (huRBL clone RS-ATL8 transfected with α/β/γ subunits of the human high-affinity IgE receptor (FcεRI)) kindly provided by Prof. Ryosuke Nakamura [56]. Cells were pre-incubated overnight with patient serum heat-inactivated at 55 °C for 25 min [57] diluted 1:10 in MEM supplemented with 10% heat-inactivated fetal bovine serum (HI FBS), 100 U/mL penicillin–streptomycin, 0.2 mM L-glutamine, 0.2 mg/mL hygromycin B, and 0.2 mg/mL geneticin (Thermo Fisher Scientific). Six different allergen concentrations (1000, 100, 10, 1, 0.1, and 0.01 ng/mL) from rAmb a 6; Par j 2, kindly provided by Prof. Rudolf Valenta [42]; and natural Amb a 1.01 (nAmb a 1.01) as a benchmark kindly provided by Dr. Frank Stolz were used to test allergenic activity. The assay was performed with minor modifications from the previously described assay [58], namely exposure to the allergen for 1 h at 37 °C, 5% CO_2_. The release of ß-hexosaminidase was detected using 4-Muc (Sigma Aldrich) detection measured at 360 nm excitation/465 nm emission on a microplate reader (Varioskan™ LUX, Thermo Fisher Scientific) and degranulation was expressed as the percentage from total mediator release (cells after addition of 10% Triton-X for cell disruption). The figures display the percentage of total ß-hexosaminidase release relative to complete cell lysis using Triton-X as the median with 95% confidence interval (CI) of triplicate measurements.

### 4.8. Association with Clinical Symptoms and Statistical Analysis

ELISA ODs and sIgE levels were tested for normal distribution using a Shapiro–Wilk test. ODs obtained for rAmb a 6 in ELISA were correlated with the values in ImmunoCAP using Spearman correlation. The association of reactivity (positive–negative) towards rAmb a 6 with certain symptoms, diagnosis, and sensitization to other allergen sources was tested using a Fisher exact test. A Mann–Whitney U-test was used to compare sIgE levels between patients with certain symptom types (nasal, ocular, asthma-like, or skin symptoms). The IgE levels were also correlated with the duration of allergic disease using Spearman correlation. The sIgE values were compared in a Friedman test and then pairwise in a Wilcoxon test first for all patients, then only for those who were rAmb a 6-positive in ImmunoCAP, and also separately depending on the number of sensitizations. The differences in sIgE levels for all of the aforementioned allergens were evaluated between different levels of sensitization (mono-, oligo-, and polysensitized) using a Kruskal–Wallis test. All statistics were performed using IBM SPSS (IBM, Chicago, IL, USA) and differences with *p* ≤ 0.05 were considered significant.

## 5. Conclusions

The non-specific lipid transfer protein from common ragweed Amb a 6 was successfully produced as a recombinant protein in an insect cell expression system. The recombinant allergen bound IgE in 30% of ragweed pollen-allergic patients included in this study. Basophil activation experiments revealed that Amb a 6 has a high allergenic activity and thus seems to be clinically relevant and comparable to Amb a 1 in mediator release. The results indicate that Amb a 6 is a ragweed pollen-specific allergen without relevant IgE cross-reactivity to other pollen and plant food nsLTPs. First, the sequence identity of Amb a 6 to almost all known allergenic plant nsLTPs was below 40%, beyond a level permitting meaningful cross-reactivity. Second, rAmb a 6 did not cross-react with the major nsLTP allergen from *Parietaria judaica*, Par j 2, when tested in basophil activation assays. Third, Amb a 6-sensitized patients did not show relevant symptoms of plant-food allergy. Accordingly, Amb a 6 seems to be a genuine ragweed pollen allergen which is important for the molecular diagnosis of ragweed pollen allergy. Furthermore, Amb 6 may be considered an important allergen to be included in molecular AIT vaccines for the treatment of ragweed pollen allergy.

## Figures and Tables

**Table 1:** Inhibition of IgE binding towards rAmb a 6 by rabbit rAmb a 6-specific serum. Rabbit rAmb a 4-specific serum and pre-immune serum were used as negative controls.

Patient Number	Coating Using rAmb a 6					
	rAmb a 6		Inhib. (%)	rAmb a 4		Inhib. (%)
	Pre-Imm *	Imm *		Pre-Imm *	Imm *	
29	0.353	0.082	76.8%	0.352	0.350	0.5%
55	0.328	0.055	83.2%	0.318	0.312	2.0%
59	1.311	0.130	90.1%	1.212	1.161	4.2%
68	0.143	0.089	37.4%	0.139	0.136	1.9%
82	1.903	0.087	95.4%	1.836	1.786	2.8%
85	0.273	0.074	73.1%	0.258	0.252	2.1%
103	3.536	0.175	95.1%	3.308	3.251	1.7%
105	2.175	0.114	94.8%	1.988	1.887	5.1%
114	0.359	0.104	71.1%	0.334	0.351	0%
128	0.149	0.082	44.9%	0.144	0.146	0%
129	0.536	0.109	79.7%	0.529	0.547	0%
148	0.116	0.058	49.9%	0.120	0.117	2.9%
**Mean**			**74.3%**			**1.9%**

* ODs in ELISA after pre-incubation with rabbit pre-immune serum (Pre-imm) and rabbit rAmb a 6 or rAmb a 4 specific immune serum (Imm).

## Data Availability

All data are contained within this article and/or in the Supplementary Materials.

## Supplementary Materials

The following supporting information can be downloaded at: https://www.mdpi.com/article/10.3390/ijms25126513/s1.

## References

[1] Arbes S.J., Gergen P.J., Elliott L., Zeldin D.C. (2005). Prevalences of Positive Skin Test Responses to 10 Common Allergens in the US Population: Results from the Third National Health and Nutrition Examination Survey. J. Allergy Clin. Immunol..

[2] Lake I.R., Jones N.R., Agnew M., Goodess C.M., Giorgi F., Hamaoui-Laguel L., Semenov M.A., Solmon F., Storkey J., Vautard R. (2017). Climate Change and Future Pollen Allergy in Europe. Environ. Health Perspect..

[3] Schaffner U., Steinbach S., Sun Y., Skjøth C.A., de Weger L.A., Lommen S.T., Augustinus B.A., Bonini M., Karrer G., Šikoparija B. (2020). Biological Weed Control to Relieve Millions from Ambrosia Allergies in Europe. Nat. Commun..

[4] Thibaudon M., Hamberger C., Guilloux L., Massot R. (2010). Ragweed Pollen in France: Origin, Diffusion, Exposure. Eur. Ann. Allergy Clin. Immunol..

[5] Makra L., Juhász M., Béczi R., Borsos E. (2005). The History and Impacts of Airborne Ambrosia (Asteraceae) Pollen in Hungary. Grana.

[6] Cecchi L., D’Amato G., Ayres J.G., Galan C., Forastiere F., Forsberg B., Gerritsen J., Nunes C., Behrendt H., Akdis C. (2010). Projections of the Effects of Climate Change on Allergic Asthma: The Contribution of Aerobiology. Allergy Eur. J. Allergy Clin. Immunol..

[7] Ozaslan C., Onen H., Farooq S., Gunal H., Akyol N. (2016). Common Ragweed: An Emerging Threat for Sunflower Production and Human Health in Turkey. Weed Biol. Manag..

[8] Bullock J., Chapman D., Schaffer S., Roy D., Girardello M., Haynes T., Beal S., Wheeler B., Dickie I., Phang Z. (2010). Assessing and Controlling the Spread and the Effects of Common Ragweed in Europe.

[9] Leru P.M., Matei D., Ianovici N. (2015). Health Impact of *Ambrosia artemisiifolia* Reflected By Allergists Practice in Romania. A Questionnaire—Based Survey. Ann. West Univ. Timişoara Ser. Biol..

[10] Leru P.M., Eftimie A.M., Anton V.F., Thibaudon M. (2019). Five-Year Data on Pollen Monitoring, Distribution and Health Impact of Allergenic Plants in Bucharest and the Southeastern Region of Romania. Medicina.

[11] Leru P.M., Anton V.F., Eftimie A.M., Stefanut S. (2022). Biologic Pollution Due to Ambrosia (Ragweed) Pollen in Urban Environment of Bucharest. Int. J. Environ. Res. Public Health.

[12] Gaudeul M., Giraud T., Kiss L., Shykoff J.A. (2011). Nuclear and Chloroplast Microsatellites Show Multiple Introductions in the Worldwide Invasion History of Common Ragweed, *Ambrosia artemisiifolia*. PLoS ONE.

[13] Newhouse C.P., Levetin E. (2004). Correlation of Environmental Factors with Asthma and Rhinitis Symptoms in Tulsa, OK. Ann. Allergy Asthma Immunol..

[14] Ziska L.H., George K., Frenz D.A. (2007). Establishment and Persistence of Common Ragweed (*Ambrosia Artemisiifolia* L.) in Disturbed Soil as a Function of an Urban-Rural Macro-Environment. Glob. Chang. Biol..

[15] El Kelish A., Zhao F., Heller W., Durner J., Winkler J.B., Behrendt H., Traidl-Hoffmann C., Horres R., Pfeifer M., Frank U. (2014). Ragweed (*Ambrosia artemisiifolia*) Pollen Allergenicity: SuperSAGE Transcriptomic Analysis upon Elevated CO_2_ and Drought Stress. BMC Plant Biol..

[16] Chen K.-W., Marusciac L., Tamas P.T., Valenta R., Panaitescu C. (2018). Ragweed Pollen Allergy: Burden, Characteristics, and Management of an Imported Allergen Source in Europe. Int. Arch. Allergy Immunol..

[17] Tamaș T.P., Buzan M.R., Zbîrcea L.E., Cotarcă M.D., Grijincu M., Păunescu V., Panaitescu C., Chen K.W. (2023). Ragweed Major Allergen Amb a 11 Recombinant Production and Clinical Implications. Biomolecules.

[18] Buzan M.R., Zbîrcea L.E., Gattinger P., Babaev E., Stolz F., Valenta R., Păunescu V., Panaitescu C., Chen K.W. (2022). Complex IgE Sensitization Patterns in Ragweed Allergic Patients: Implications for Diagnosis and Specific Immunotherapy. Clin. Transl. Allergy.

[19] Zbîrcea L.E., Buzan M.R., Grijincu M., Babaev E., Stolz F., Valenta R., Păunescu V., Panaitescu C., Chen K.W. (2023). Relationship between IgE Levels Specific for Ragweed Pollen Extract, Amb a 1 and Cross-Reactive Allergen Molecules. Int. J. Mol. Sci..

[20] Caraballo L., Valenta R., Puerta L., Pomés A., Zakzuk J., Fernandez-Caldas E., Acevedo N., Sanchez-Borges M., Ansotegui I., Zhang L. (2020). The Allergenic Activity and Clinical Impact of Individual IgE-Antibody Binding Molecules from Indoor Allergen Sources. World Allergy Organ. J..

[21] Caraballo L., Valenta R., Acevedo N., Zakzuk J. (2021). Are the Terms Major and Minor Allergens Useful for Precision Allergology?. Front. Immunol..

[22] Trifonova D., Curin M., Riabova K., Karsonova A., Keller W., Grönlund H., Käck U., Konradsen J.R., van Hage M., Karaulov A. (2023). Allergenic Activity of Individual Cat Allergen Molecules. Int. J. Mol. Sci..

[23] Edqvist J., Blomqvist K., Nieuwland J., Salminen T.A. (2018). Plant Lipid Transfer Proteins: Are We Finally Closing in on the Roles of These Enigmatic Proteins?. J. Lipid Res..

[24] Palacin A., Varela J., Quirce S., Del Pozo V., Tordesillas L., Barranco P., Fernandez-Nieto M., Sastre J., Diaz-Perales A., Salcedo G. (2009). Recombinant Lipid Transfer Protein Tri a 14: A Novel Heat and Proteolytic Resistant Tool for the Diagnosis of Baker’s Asthma. Clin. Exp. Allergy.

[25] Pastorello E.A., Robino A.M. (2004). Clinical Role of Lipid Transfer Proteins in Food Allergy. Mol. Nutr. Food Res..

[26] Asero R., Mistrello G., Amato S. (2011). The Nature of Melon Allergy in Ragweed-Allergic Subjects: A Study of 1000 Patients. Allergy Asthma Proc..

[27] Fernández-Rivas M., González-Mancebo E., Rodríguez-Pérez R., Benito C., Sánchez-Monge R., Salcedo G., Alonso M.D., Rosado A., Tejedor M.A., Vila C. (2003). Clinically Relevant Peach Allergy Is Related to Peach Lipid Transfer Protein, Pru p 3, in the Spanish Population. J. Allergy Clin. Immunol..

[28] Sala-Cunill A., Bartra J., Dalmau G., Tella R., Botey E., Raga E., Valero A. (2013). Prevalence of Asthma and Severity of Allergic Rhinitis Comparing 2 Perennial Allergens: House Dust Mites and Parietaria Judaica Pollen. J. Investig. Allergol. Clin. Immunol..

[29] Ridolo E., Barone A., Ottoni M., Peveri S., Montagni M., Nicoletta F. (2023). Factors and Co-Factors Influencing Clinical Manifestations in NsLTPs Allergy: Between the Good and the Bad. Front. Allergy.

[30] Lombardero M., García-Sellés F.J., Polo F., Jimeno L., Chamorro M.J., García-Casado G., Sánchez-Monge R., Díaz-Perales A., Salcedo G., Barber D. (2004). Prevalence of Sensitization to Artemisia Allergens Art v 1, Art v 3 and Art v 60 KDa. Cross-Reactivity among Art v 3 and Other Relevant Lipid-Transfer Protein Allergens. Clin. Exp. Allergy.

[31] Asero R., Brusca I., Cecchi L., Pignatti P., Pravettoni V., Scala E., Uasuf C.G., Villalta D. (2022). Why Lipid Transfer Protein Allergy Is Not a Pollen-Food Syndrome: Novel Data and Literature Review. Eur. Ann. Allergy Clin. Immunol..

[32] Zhao L., Fu W., Gao B., Liu Y., Wu S., Chen Z., Zhang X., Wang H., Feng Y., Wang X. (2020). Variation in IgE Binding Potencies of Seven Artemisia Species Depending on Content of Major Allergens. Clin. Transl. Allergy.

[33] Roebber M., Hussain R., Klapper D.G., Marsh D.G. (1983). Isolation and Properties of a New Short Ragweed Pollen Allergen, Ra6. J. Immunol..

[34] Marsh D.G., Freidhoff L.R., Ehrlich-kautzky E., Bias W.B., Roebber M. (1987). Immune Responsiveness to *Ambrosia artemisiifolia* (Short Ragweed) Pollen Allergen Amb a VI (Ra6) Is Associated with HLA-DR5 in Allergic Humans. Immunogenetics.

[35] Curin M., Garib V., Valenta R. (2017). Single Recombinant and Purified Major Allergens and Peptides: How They Are Made and How They Change Allergy Diagnosis and Treatment. Ann. Allergy Asthma Immunol..

[36] Hiller K.M., Lubahn B.C., Klapper D.G. (1998). Cloning and Expression of Ragweed Allergen Amb a 6. Scand. J. Immunol..

[37] Salminen T.A., Blomqvist K., Edqvist J. (2016). Lipid Transfer Proteins: Classification, Nomenclature, Structure, and Function. Planta.

[38] Missaoui K., Gonzalez-Klein Z., Pazos-Castro D., Hernandez-Ramirez G., Garrido-Arandia M., Brini F., Diaz-Perales A., Tome-Amat J. (2022). Plant Non-Specific Lipid Transfer Proteins: An Overview. Plant Physiol. Biochem..

[39] ProtParam Tool. https://web.expasy.org/protparam/.

[40] SWISS-MODEL Online Tool. https://swissmodel.expasy.org/.

[41] Database of Protein Domains, Families and Functional Sites. https://prosite.expasy.org/.

[42] Dorofeeva Y., Colombo P., Blanca M., Mari A., Khanferyan R., Valenta R., Focke-Tejkl M. (2019). Expression and Characterization of Recombinant Par j 1 and Par j 2 Resembling the Allergenic Epitopes of Parietaria Judaica Pollen. Sci. Rep..

[43] Pokoj S., Lauer I., Fötisch K., Himly M., Mari A., Enrique E., Miguel-Moncin M.D.M.S., Lidholm J., Vieths S., Scheurer S. (2010). Pichia Pastoris Is Superior to *E. coli* for the Production of Recombinant Allergenic Non-Specific Lipid-Transfer Proteins. Protein Expr. Purif..

[44] Martín-Pedraza L., Wangorsch A., Bueno-Diaz C., de las Heras M., Scheurer S., Cuesta-Herranz J., Villalba M. (2020). 2S Albumins and NsLTP Are Involved in Anaphylaxis to Pizza Sauce: IgE Recognition before and after Allergen Processing. Food Chem..

[45] Barber D., De La Torre F., Feo F., Florido F., Guardia P., Moreno C., Quiralte J., Lombardero M., Villalba M., Salcedo G. (2008). Understanding Patient Sensitization Profiles in Complex Pollen Areas: A Molecular Epidemiological Study. Allergy Eur. J. Allergy Clin. Immunol..

[46] Aalberse R.C. (2000). Structural Biology of Allergens. J. Allergy Clin. Immunol..

[47] Skypala I.J., Cecchi L., Shamji M.H., Scala E., Till S. (2019). Lipid Transfer Protein Allergy in the United Kingdom: Characterization and Comparison with a Matched Italian Cohort. Allergy.

[48] Westman M., Asarnoj A., Ballardini N., Andersson N., Kiewiet M.B.G., Borres M.P., Apostolovic D., Kull I., Bergström A., Melén E. (2022). Alpha-Gal Sensitization among Young Adults Is Associated with Male Sex and Polysensitization. J. Allergy Clin. Immunol. Pract..

[49] WHO/IUIS Allergen Nomenclature SubCommittee Allergen Nomenclature. IUIS Database. https://allergen.org/.

[50] NIH Basic Local Alignment Search Tool. https://blast.ncbi.nlm.nih.gov.

[51] Tamura K., Stecher G., Kumar S. (2021). MEGA11: Molecular evolutionary genetics analysis version 11. Mol. Biol. Evol..

[52] Grijincu M., Hutu I., Weber M., Babaev E., Stolz F., Valenta R., Paunescu V., Panaitescu C., Chen K.-W. (2023). Physicochemical and Immunological Characterization of Amb a 12, a Novel Ragweed (*Ambrosia artemisiifolia*) Pollen Allergen. Mol. Immunol..

[53] Campana R., Vrtala S., Maderegger B., Dall’Antonia Y., Zafred D., Blatt K., Herrmann H., Focke-Tejkl M., Swoboda I., Scheiblhofer S. (2011). Altered IgE Epitope Presentation: A Model for Hypoallergenic Activity Revealed for Bet v 1 Trimer. Mol. Immunol..

[54] Sreerama N., Woody R.W. (2000). Estimation of Protein Secondary Structure from Circular Dichroism Spectra: Comparison of CONTIN, SELCON, and CDSSTR Methods with an Expanded Reference Set. Anal. Biochem..

[55] Lungu B., Georgescu O., Tudor B., Buzan R., Grijincu M., Cotarca M., Panaitescu C., Chen K.-W., Torda I., Mircu C. (2022). Study of Some Factors Associated with Polyclonal Antibody Production in Rabbit. Eximia.

[56] Nakamura R., Uchida Y., Higuchi M., Nakamura R., Tsuge I., Urisu A., Teshima R. (2010). A Convenient and Sensitive Allergy Test: IgE Crosslinking-Induced Luciferase Expression in Cultured Mast Cells. Allergy Eur. J. Allergy Clin. Immunol..

[57] Takagi K., Nakamura R., Teshima R., Sawada J.I. (2003). Application of Human FcεRI α-Chain-Transfected RBL-2H3 Cells for Estimation of Active Serum IgE. Biol. Pharm. Bull..

[58] Gieras A., Focke-Tejkl M., Ball T., Verdino P., Hartl A., Thalhamer J., Valenta R. (2007). Molecular Determinants of Allergen-Induced Effector Cell Degranulation. J. Allergy Clin. Immunol..

