# Supplementary material for: Non-Specific Lipid Transfer Protein Amb a 6 Is a Source-Specific Important Allergenic Molecule in Ragweed Pollen
Source: Int J Mol Sci. 2024 Jun 13;25(12):6513. doi: 10.3390/ijms25126513 (PMC11204090; doi:10.3390/ijms25126513)
Supplement: Supplementary file 1 [file ijms-25-06513-s001.zip › Figure S1. rAmb a 6-specific IgG after rabbit immunization.pdf]

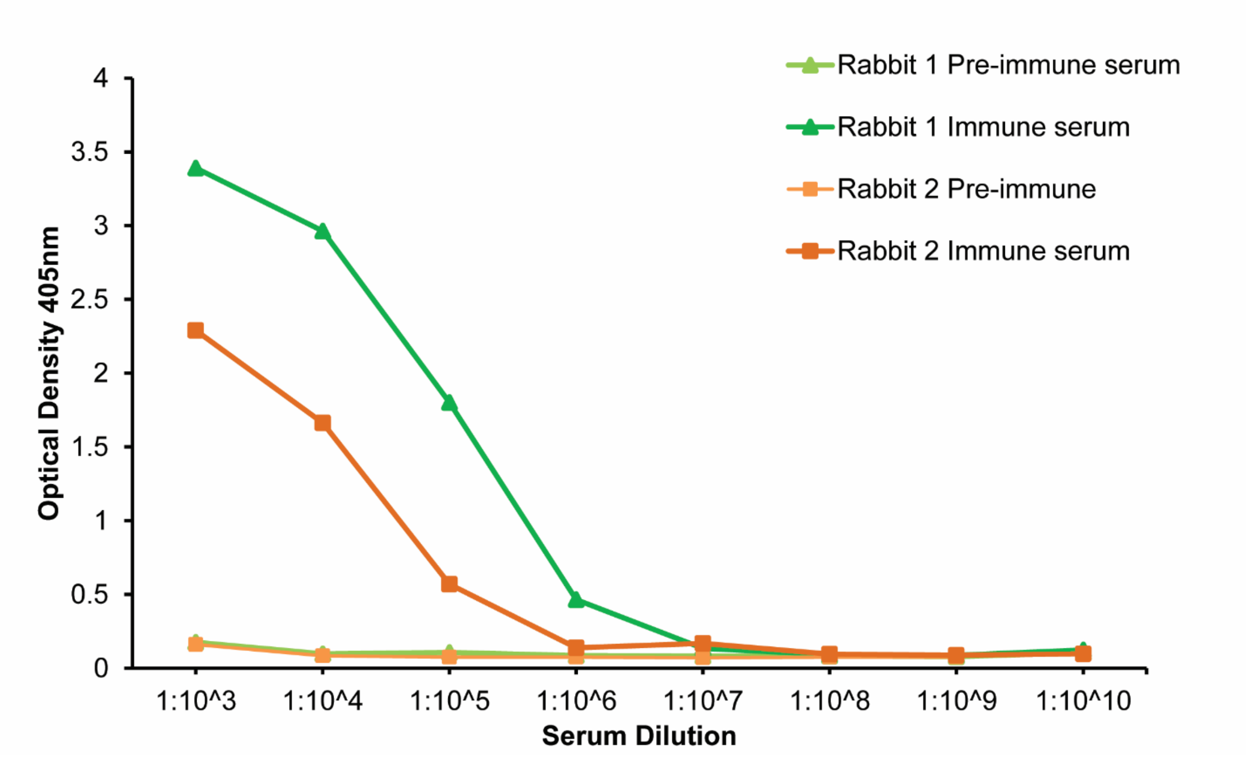

Figure S1. rAmb a 6-specific IgG after rabbit immunization. Rabbit IgG response towards rAmb a 6 before immunization (pre-immune) and after immunization (immune serum) determined in ELISA. IgG response in serum from rabbit 1 is shown in green, IgG response in serum from rabbit 2 in orange, pre-immune serum is displayed in lighter shades of green and orange. Optical density was measured 15 min after substrate addition.
